# Dose‐Dependent Effects of Clinoptilolite Supplementation on Sperm Quality and Oxidative Stress During Canine Semen Cryopreservation

**DOI:** 10.1002/vms3.71128

**Published:** 2026-08-05

**Authors:** Nurdan Coşkun, Fikret Karaca

**Affiliations:** ^1^ Faculty of Veterinary Medicine Department of Reproduction and Artificial Insemination Hatay Mustafa Kemal University Hatay Turkey

**Keywords:** canine semen, clinoptilolite, oxidative stress, semen cryopreservation, sperm quality

## Abstract

**Background:**

Cryopreservation induces oxidative stress, leading to reduced semen quality in canine semen. Clinoptilolite (CLP), a natural zeolite, has potential antioxidant properties that may improve post‐thaw sperm parameters.

**Objectives:**

This study aimed to evaluate the dose‐dependent effects of CLP supplementation in semen extenders on sperm quality and oxidative stress in dogs.

**Methods:**

Semen was collected from four adult dogs by manual manipulation, divided into five equal aliquots, and diluted at a ratio of 1:3 (v/v) with a Tris–citrate extender containing 0% (control), 1%, 2%, 3%, or 4% CLP. Samples were cooled to 5°C within 45 min, equilibrated for 2 h in 0.25 mL straws, frozen in liquid nitrogen vapour, and stored in liquid nitrogen. Samples were thawed at 37°C for 30 s. Post‐thaw sperm quality was evaluated together with oxidative stress markers, including total antioxidant status (TAS), total oxidant status (TOS), and oxidative stress index (OSI).

**Results:**

The 2% CLP group showed the highest post‐thaw sperm quality, whereas the lowest performance was observed in the 4% CLP group. Additionally, the 2% CLP group exhibited a more favourable oxidative profile, with higher TAS and lower TOS and OSI values than all other groups.

**Conclusions:**

CLP supplementation exerted dose‐dependent effects on sperm quality and oxidative balance in canine semen cryopreservation. A 2% CLP concentration appears to be the most effective for improving post‐thaw sperm quality.

## Graphical abstract

Clinoptilolite supplementation in canine semen extenders showed dose‐dependent effects during cryopreservation, with 2% yielding optimal post‐thaw sperm quality. Higher concentrations impaired sperm parameters, likely due to oxidative imbalance, highlighting the importance of precise dosing to enhance cryosurvival.

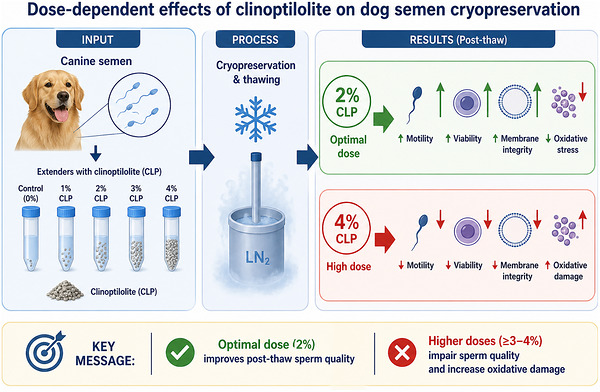

## 1 Introduction

Compared with other domestic animal species, relatively few studies have investigated canine semen preservation. Semen from dogs of high genetic value is routinely transported nationally and internationally, particularly in frozen form, for use in artificial insemination programmes. Canine semen banks have been established in the United States and many European countries to facilitate the use of genetically valuable dogs. Further research is needed to reduce the detrimental effects associated with short‐ and long‐term sperm preservation methods have on spermatozoa. For many years, studies have focused on identifying canine semen characteristics, optimizing semen extenders and freezing protocols, improving storage conditions, and maximizing fertility following artificial insemination. Currently, various extenders are currently used as sperm diluents for the short‐term or long‐term preservation of dog sperm. For canine semen cryopreservation, solutions such as Krebs‐Ringer, sodium citrate, lactose, and milk have been widely used. However, in recent years, zwitterionic buffers like Tris, Tes, Bes, Hepes, Mes, Pipes, and Mops have been intensively employed. Additionally, canine semen can also be stored for long periods using commercial preparations such as Laiciphos, Biociphos, Biladyl, and Triladyl (Tosun and Uysal 2007). During storage, sperm damage is due to physical and biochemical stressors on some components (Khalil et al. 2018). To protect the sperm cells from these impacts, some antioxidant substances are added to extenders, and the composition of extenders should be optimized for each species (Mohammed et al. 2021).

Cryopreservation increases the levels of intracellular reactive oxygen species (ROS), leading to oxidative stress. Oxidative stress compromises fertilization capacity by affecting sperm viability, plasma membrane, and acrosomal integrity (Aitken and Baker 2004; Ball et al. 2001). Oxidative stress also causes DNA damage (Coyan et al. 2012). During the processing of sperm, cryoprotective and antioxidant substances are added to the diluents to counteract such adverse effects, and the diluent composition and protocol should be optimized and developed according to the animal species. To protect spermatozoa from ROS‐induced oxidative stress and lipid peroxidation, exogenous antioxidants are added to semen extenders to support the endogenous antioxidant defence system, thereby preserving sperm motility, viability, membrane integrity, capacitation, and fertilizing ability (Henkel et al. 2005).

Zeolites are aluminosilicate minerals in natural or synthetic forms (Alberti and Bein 1996). Zeolites were discovered in 1756 by Baron Axel Fredrick Cronstedt (Mumpton and Fishman 1977). Each zeolite has uniformly distributed nanopores and morphology irrespective of its particle size. Synthetic forms of zeolites have also been used as adjuvants and antimicrobials for medicine (Mohammed et al. 2021; Salah et al. 2019). Some forms of zeolites have been used in antifungal, antibacterial, and antidiarrheal applications (Kraljevic Pavelic et al. 2018). Previous studies also indicated zeolites have antioxidant properties and are used in veterinary medicine (Mohammed et al. 2021; Wu et al. 2013). Zeolites have been extensively used as feed additives in farm animals to positively influence growth and reproductive performance and are considered chemically inert and generally safe for animal use (Ivkovic et al. 2004; Papaioannou et al. 2005; Valpotić et al. 2017). Clinoptilolite (CLP) is a naturally occurring three‐dimensional aluminosilicate mineral and is the most widely used zeolite in veterinary medicine (Pavelic et al. 2001). Zeolite can adsorb toxic molecules and participate in catalysis and dehydration‐rehydration processes (Mumpton 1999; Oguz and Kurtoglu 2000; Schneider et al. 2017). CLP is a great source of silicon in orthosilicic acid, which protects the body from heavy metals (Martin 2013). CLP could also intoxicate some species (Papaioannou et al. 2005). Limited information is available about the effects of zeolites on reproductive activity. It has been shown that CLP has antibacterial, antioxidant, and immunomodulatory effects in various mammalian species (Mastinu et al. 2019; Kraljevic Pavelic et al. 2018; Valpotić et al. 2017). There is no report on the addition of CLP in semen extenders regarding the effects on spermatological characteristics and antioxidant status of cryopreserved semen in dogs. Therefore, the present study aimed to evaluate the impact of different CLP ratios added to sperm extenders on sperm quality and oxidative stress in the long‐term storage of dog sperm.

## 2 Materials and Methods

The study was conducted with four dogs aged 2–5 years. The Hatay Mustafa Kemal University Scientific Ethical Committee approved the experiments (decision number 2022/03‐05).

### 2.1 Sperm Collection

Semen was collected using the massage method (Yildiz et al. 2000) twice a week for a total of 10 repetitions. In the collection of sperm, one person held the sperm cup, while the other approached the dog from the right side and started massaging the bulbus glandis area with a vaselined hand. After the bulbus glandis area in the dog swelled, the massage continued from behind the bulbus glandis with the completely retracted prepuce. The penis was directed ventrally to facilitate the dog's ejaculation. The second part, which was dense with spermatozoa, was collected, and the ejaculates with normospermia characteristics were used in evaluations. After the sperm was collected, an antibiotic ointment was applied to the penis, and the procedure was completed by placing the penis back into the prepuce. Then, the semen was transferred to the laboratory after collection, and the temperature of the laboratory was 25–30°C.

### 2.2 Preparation of Clinoptilolite Solutions

CLP stock solution was prepared by dissolving 4 g of CLP in 100 mL of distilled water. The stock solution was thoroughly mixed to ensure homogeneity and subsequently used to obtain the desired working concentrations (1%, 2%, 3%, and 4%) by appropriate dilution.

The CLP used in this study was obtained from a commercial source (Rota Mining Corporation, Türkiye; 10 µm particle size). Zeolite supplementation (∼1%) has been reported to improve post‐thaw sperm quality, particularly in terms of motility, viability, and membrane integrity (Mohammed et al. 2019; Mohammed et al. 2021). Considering these findings, together with our preliminary observations indicating limited effects below 1% and adverse effects above 4%, CLP concentrations between 1% and 4% were selected to evaluate dose‐dependent effects in canine semen cryopreservation.

### 2.3 Dilution of the Semen

Semen samples were diluted at a ratio of 1:3 (v/v) to a final concentration of 100 × 10^6^ spermatozoa/mL using a Tris–citrate extender (240 mM Tris, 63 mM citric acid, 8% [v/v] glycerol, 20% [v/v] egg yolk, and 70 mM fructose). The experimental groups were as follows: control (Tris–citrate) and Tris–citrate supplemented with 1%, 2%, 3%, and 4% CLP.

### 2.4 Freezing and Thawing of Semen

Only ejaculates exhibiting at least 70% motility and less than 10% dead spermatozoa were included in the study. Semen samples were first diluted with Tris–citrate extender containing the respective CLP concentrations (control, 1%, 2%, 3%, and 4%). Following dilution, samples were gradually cooled from room temperature to 5°C over 45 min. The cooled samples were then loaded into 0.25‐mL straws and equilibrated at 5°C for 2 h. After equilibration, the straws were placed horizontally at a distance of 4 cm above the surface of liquid nitrogen and exposed to nitrogen vapour for 20 min. Subsequently, the straws were plunged directly into liquid nitrogen (−196°C) and stored until further analysis. The straws were thawed in a water bath at 37°C for 30 s, based on the method described by Yildiz et al. (2000) with slight modifications, and spermatological parameters were assessed as described in the following sections.

### 2.5 Motility

Spermatozoon motility was determined on a heating stage at 37°C placed on the phase contrast microscope after dilution, equilibration, and thawing. Motility was examined in at least three microscope fields at 400× magnification. The ratio of spermatozoa moving smoothly in one direction to all spermatozoa in the same field was expressed as a percentage (Yildiz et al. 2000).

### 2.6 Abnormal Spermatozoa

The percentage of abnormal spermatozoa was determined using eosin–nigrosin staining according to Hafez and Hafez (2000), with slight modifications after dilution, equilibration, and thawing. A small drop of sample was placed on a clean glass slide, and two drops of eosin‐nigrosin stain were added and gently mixed. A smear was then prepared and air‐dried. The slides were examined under a light microscope at 1000× magnification using immersion oil. In each preparation, a total of 400 spermatozoa were evaluated from a selected field, and the percentage of abnormal spermatozoa was calculated.

### 2.7 Sperm Viability

After dilution, equilibration, and thawing, sperm viability was assessed using the Live/Dead Kit (L7011; Invitrogen, Carlsbad, CA, USA) according to the manufacturer's protocol. In short, SYBR‐14 was dissolved in dimethyl sulfoxide (DMSO) at a 1/10 ratio, transferred to an Eppendorf tube in 10 µL volumes, and stored at −20°C. The Propidium iodide (PI) stock solution was as follows: 1 mg propidium iodide dissolved in 1 mL phosphate buffer (PBS) was transferred to an Eppendorf tube in 10 µL volumes and stored at −20°C. For analysis, the semen samples at +37°C were transferred to an Eppendorf tube, and 300 µL of PBS was added. Then, 6 µL of SYBR‐14 stock solution and 2.5 µL of PI stock solution were added to 30 µL of the sample, and it was incubated in a water bath at +37°C for 15 min. Then, 5 µL of the solution was placed on a slide, covered with a coverslip, and evaluated under a fluorescent microscope. Those with light green‐stained heads were recorded as live, while those with red heads were recorded as dead.

### 2.8 Plasma Membrane Integrity

The Hypo‐Osmotic Swelling (HOS) test was applied to determine membrane integrity in sperm samples after dilution, equilibration, and thawing. The HOS test solution was prepared by dissolving 1.1 g of fructose and 0.55 g of sodium citrate in 100 mL of distilled water to achieve a final concentration of 100 mOsm/L. Note that 100 µL of the 100 mOsm HOS solution at 37°C in a water bath was taken, and 10 µL from the sperm sample was added on top. After incubating this mixture at 37°C for 60 min, a drop was taken onto a slide, a smear was made and placed on a heating plate. The prepared smears were examined under a microscope (×400), 200 cells were counted, and the percentage of spermatozoa responding to the HOS test was determined by considering the bends and swellings in the tail described as Nur et al. (2005).

### 2.9 Acrosome Integrity

**FIGURE 1:**
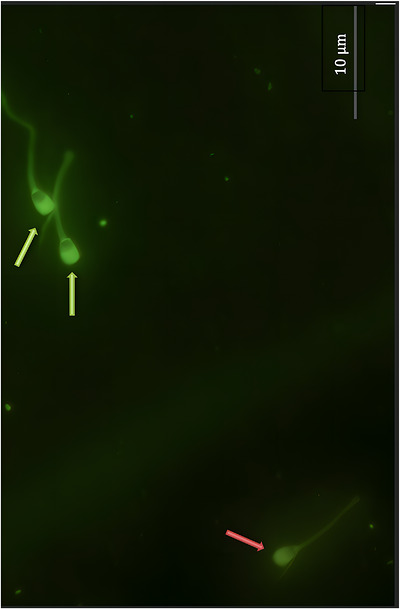
Assessment of acrosome integrity using FITC–PSA staining. Green arrows indicate spermatozoa with intact acrosomes, whereas the red arrow indicates a spermatozoon with a damaged acrosome.

Acrosome integrity was assessed using FITC‐PSA (L0770, Sigma) according to Nur et al. (2010) after thawing. Briefly, 20 mL of diluted semen was resuspended in 500 mL PBS and centrifuged at 2000 rpm for 20 min; the supernatant was then discarded. The spermatozoa pellet was resuspended in 250 mL PBS. One drop of resuspended spermatozoa was smeared on a glass microscope slide and air dried. Air‐dried slides were fixed with acetone at 4°C for 10 min, and the slides were covered with FITC PSA solution (50 mg/mL in PBS solution) in the dark for 30 min. The stained slides were rinsed with PBS solution, covered with glycerol, and examined under a fluorescence microscope. At least 100 spermatozoa per smear were evaluated for acrosome integrity under a fluorescent microscope (Figure 1).

### 2.10 Oxidative Stress

In post‐thaw sperm straws, total antioxidant status (TAS) and total oxidant status (TOS) levels were measured using commercial kits (Rel Assay Diagnostics, Turkey) according to the manufacturer's instructions, based on the methods described by Erel (2004, 2005) with an autoanalyzer. The results were expressed as mmol Trolox equivalent/L for TAS. The assay was calibrated with hydrogen peroxide, and the results were expressed in terms of micromolar hydrogen peroxide equivalent per litre for TOS (µmol H_2_O_2_ equivalent/L). The ratio of TOS to TAS was accepted as the oxidative stress index (OSI). For calculation, the OSI value was calculated according to the following formula: OSI (Arbitrary Unit) = TOS (µmol H_2_O_2_ equivalent/L)/TAS (µmol Trolox equivalent/L). The OSI was calculated as the ratio of TOS to TAS after converting TAS values from mmol/L to µmol/L. For ease of interpretation, the resulting OSI values were multiplied by 1000 and expressed as arbitrary units (AU).

### 2.11 Statistical Analysis

The mean values and standard errors of the data obtained in the study were calculated. Statistical analyses were evaluated with the SPSS 23.0 package program. A one‐way analysis of variance (ANOVA) test was used to evaluate the data. The Duncan test was used to indicate the importance of the difference between groups as a result of the analysis.

## 3 Results

In the evaluation conducted after dilution in Table 1, motility and membrane integrity were found to be the highest in the 2% and 3% CLP groups, while the lowest values were determined in the 4% CLP added group (*p* < 0.05). In the control and 1% CLP added groups, motility and membrane integrity were found to be similar. The dead spermatozoon rate after dilution was found to be the highest in the 4% CLP group and the lowest in the 1% and 2% CLP added groups. The dead spermatozoon rate observed in the control group was higher than in the other test groups, except for the 4% CLP group. In terms of the abnormal spermatozoon rate, the highest value was recorded in the control and 4% CLP groups, while the lowest was in the 2% CLP group (*p* < 0.05).

**TABLE 1:** Post‐dilution spermatological characteristics of canine semen in different extender groups (mean ± standard error).

Groups	Motility (%)	Membrane integrity (%)	Dead spermatozoa rates (%)	Abnormal spermatozoa rates (%)
Control	80 ± 2.50b	84 ± 2.55b	15 ± 1.46b	11 ± 0.60a
1% CLP	80 ± 2.50b	82 ± 3.66b	5 ± 1.44d	9 ± 3.98b
2% CLP	85 ± 2.50a	88 ± 2.65a	5 ± 2.48d	4 ± 1.56d
3% CLP	85 ± 5.00a	87 ± 5.42a	7 ± 3.98c	6 ± 3.12c
4% CLP	70 ± 5.00c	71 ± 4.30c	19 ± 3.16a	12 ± 4.00a
*p* value	0.030	0.020	0.001	0.001

*Note*: Differences were considered statistically significant at *p* < 0.05. There is a statistical difference between the values indicated with different letters in the same column.

In post‐equilibration spermatozoa evaluations, motility and membrane integrity were found to be the highest in the 2% and 3% CLP groups, while the lowest was determined in the 4% CLP group. The highest dead spermatozoon rate was found in the 4% CLP group, while the lowest value was observed in the 1% and 2% CLP supplemented groups. In terms of the abnormal spermatozoon rate, the highest value after equilibration was found in the control and 4% CLP groups, while the lowest abnormal spermatozoon rate was determined in the 2% CLP group (Table 2) (*p* < 0.05).

**TABLE 2:** Post‐equilibration spermatological parameters in the different extender groups (mean ± standard error).

Groups	Motility (%)	Membrane integrity (%)	Dead spermatozoa rates (%)	Abnormal spermatozoa rates (%)
Control	75 ± 2.50b	80 ± 2.10b	20 ± 2.24b	16 ± 2.23a
1% CLP	75 ± 5.00b	78 ± 2.44b	7 ± 2.24d	12 ± 4.39b
2% CLP	80 ± 2.50a	82 ± 2.98a	7 ± 3.14d	6 ± 2.20d
3% CLP	80 ± 2.50a	82 ± 4.66a	10 ± 2.14c	9 ± 4.42c
4% CLP	60 ± 5.00c	63 ± 3.38c	23 ± 4.51a	15 ± 5.30a
*p* value	0.001	0.001	0.001	0.010

*Note*: Differences were considered statistically significant at *p* < 0.05. There is a statistical difference between the values indicated with different letters in the same column.

In Table 3, the sperm quality in the extender groups after thawing was presented. After thawing, the highest motility was observed in the 2% and 3% CLP groups, while the lowest motility was found in the 4% CLP group. The control group was found to have higher motility than the group with 1% CLP addition. In the evaluation of membrane integrity, the 2% and 3% CLP groups had the highest values after thawing, while the lowest value was found in the 4% CLP group. The control group and the group with 1% CLP addition had similar membrane integrity. After thawing, the highest dead spermatozoon rate was found in the 4% CLP group, while the lowest dead spermatozoon rate was observed in the 1% and 2% CLP groups. In terms of the abnormal spermatozoon rate, the highest value after thawing was found in the control and 4% CLP groups. The lowest abnormal spermatozoon rate was found in the 2% CLP group. After thawing, the lowest acrosome damage was recorded in the 2% CLP group, while the highest was in the 4% CLP group (*p* < 0.05). The control group was found to have a similar acrosome damage rate to the 1% and 3% CLP groups (*p* > 0.05).

**TABLE 3:** Post‐thaw spermatological parameters in the different extender groups (mean ± standard error).

Groups	Motility (%)	Membrane integrity (%)	Dead spermatozoa rates (%)	Abnormal spermatozoa rates (%)	Acrosome damage (%)
Control	40 ± 5.95b	48 ± 4.20b	30 ± 3.36b	16 ± 4.42a	8 ± 2.44b
1% CLP	35 ± 5.00c	40 ± 4.00b	17 ± 3.46d	13 ± 4.36b	8 ± 3.36b
2% CLP	45 ± 5.55a	52 ± 5.08a	16 ± 2.50d	8 ± 3.22d	4 ± 2.21c
3% CLP	45 ± 5.50a	52 ± 5.26a	25 ± 5.00c	11 ± 4.14c	6 ± 3.26bc
4% CLP	25 ± 5.00d	30 ± 2.58c	40 ± 4.65a	17 ± 5.88a	11 ± 4.86a
*p* value	0.001	0.001	0.001	0.030	0.001

*Note*: Differences were considered statistically significant at *p* < 0.05. There is a statistical difference between the values indicated with different letters in the same column.

In terms of oxidative stress determined in sperm, the highest total antioxidant ratio was found in the 2% and 3% CLP groups, while the lowest was in the 4% CLP group (*p* < 0.05). The control and 3% CLP groups were found to be similar. In terms of total oxidant status, the highest value was found in the 4% and 1% CLP groups, while the lowest value was found in the 2% CLP group (*p* < 0.05). The control and 3% CLP supplemented groups had similar TOS values. The OSI was the highest in the 1% and 4% CLP groups and the lowest in the 2% CLP group (*p* < 0.05). The control group and the 3% CLP group had similar OSI values (Table 4).

**TABLE 4:** Oxidative stress parameters in the different cryopreserved semen extender groups (mean ± standard error).

Groups	TAS (mmol Trolox eq./L)	TOS (µmol H_2_O_2_ eq./L)	OSI (AU)
Control	1.7 ± 0.39b	8 ± 2.42b	4.7 ± 1.34c
1% CLP	1.6 ± 0.50b	10 ± 4.01a	6.2 ± 2.38b
2% CLP	2.0 ± 0.22a	5 ± 2.08c	2.5 ± 0.80d
3% CLP	1.9 ± 0.25a	8 ± 3.16b	4.2 ± 1.55c
4% CLP	1.3 ± 0.89c	12 ± 3.46a	9.2 ± 3.45a
*p* value	0.010	0.010	0.012

*Note*: Differences were considered statistically significant at *p* < 0.05. There is a statistical difference between the values indicated with different letters in the same column.

## 4 Discussion

The results of the present study demonstrated a consistent dose‐dependent effect of CLP supplementation on sperm quality and oxidative stress parameters during dilution, equilibration, and cryopreservation. In general, moderate CLP concentrations (2% and 3%) resulted in improved spermatological outcomes, including higher motility and membrane integrity. Low CLP concentrations (1% and 2%) were associated with reduced proportions of dead spermatozoa. In particular, the 2% CLP group exhibited the lowest proportions of abnormal and acrosome‐damaged spermatozoa compared with the control and higher concentrations. In contrast, the 4% CLP group consistently showed the poorest sperm quality across all stages, with significantly reduced motility and membrane integrity and increased sperm damage after cryopreservation. Parallel to these findings, oxidative stress analysis indicated that the 2% CLP group exhibited lower TOS and OSI values and higher TAS levels, suggesting improved redox balance at this concentration. Conversely, the 4% CLP concentration was associated with increased oxidative stress and elevated OSI values. Overall, these findings suggest that CLP exerts a concentration‐dependent dual effect, with low‐to‐moderate supplementation improving both sperm quality and oxidative stability, while excessive levels may be detrimental to sperm function during cryopreservation.

Previous studies conducted in different animal species have reported beneficial effects of zeolite supplementation on semen quality parameters, although results vary depending on dose, form, and experimental model. Mohammed et al. (2019) reported that supplementation of rabbit semen extenders with 1% zeolite improved post‐thaw sperm quality, including motility, viability, and membrane integrity, while also reducing sperm damage and apoptosis. However, this improvement was not accompanied by enhanced total antioxidant capacity, and in fact, increased oxidative stress markers such as malondialdehyde and H_2_O_2_ were observed. Mohammed et al. (2021) demonstrated that zeolite (Z±) supplementation of semen extender similarly improved sperm motility, viability, and membrane integrity and reduced apoptotic and necrotic sperm cells after equilibration and thawing. These effects were not associated with significant changes in oxidative biomarkers, suggesting that zeolite's beneficial effects on sperm quality may occur independently of classical antioxidant mechanisms. Similarly, improvements in sperm concentration, motility, and reductions in dead and abnormal spermatozoa have been reported in roosters following CLP supplementation orally (Morsy 2018). In rats, dietary supplementation with 2%, 4%, and 6% zeolite resulted in a significantly higher proportion of abnormal spermatozoa at the 6% inclusion level, suggesting a dose‐dependent adverse effect (Tas et al. 2007). The fact that high zeolite concentrations reduce sperm quality is consistent with our study. Beneficial effects of CLP have also been observed in systemic reproductive and metabolic parameters in livestock species, including improvements in metabolic status and reproductive performance (Folnožić et al. 2019). However, despite these positive findings across species, there is currently no available study investigating the effect of CLP on semen cryopreservation in dogs, making direct comparison limited. The present results are generally consistent with previous reports indicating that low‐to‐moderate zeolite supplementation improves sperm quality, while higher concentrations may be detrimental.

The oxidative stress analysis in the present study revealed a clear dose‐dependent response of CLP supplementation in semen extenders. TAS values were the highest in the 2% and 3% CLP groups compared with the control and 1% CLP group, whereas the lowest TAS level was observed in the 4% CLP group. In contrast, TOS values were significantly reduced in the 2% CLP group, while higher values were recorded in the 1% and 4% CLP groups. Consistently, OSI values showed the most favourable redox status in the 2% CLP group, whereas the highest OSI was observed in the 4% CLP group. Collectively, the TAS, TOS, and OSI findings indicate that 2% CLP supplementation provided the most balanced oxidative status, while both insufficient (1%) and excessive (4%) concentrations were associated with a less favourable redox profile.

The mechanisms underlying the effects of CLP observed in the present study remain speculative, as no direct assessments of ROS production, lipid peroxidation, or apoptotic pathways were performed. The beneficial effects observed at moderate concentrations may be related to the physicochemical properties of CLP, particularly its adsorption capacity, ion‐exchange potential, and ability to provide trace metal ions that may contribute to the activation of antioxidant enzymes, which have been previously reported to contribute to stabilization of extracellular environment during semen processing (Kraljevic Pavelic et al. 2018; Mastinu et al. 2019). Among 140 types of natural zeolites, CLP is the most widespread. CLP is a natural mineral that has been used in veterinary and human medicine as an immunostimulant and to regulate body pH and to protect against free radical‐induced oxidative damage (Ambrozova et al. 2017; Kraljevic Pavelic et al. 2018; Šperanda et al. 2021). CLP has been shown to have antibacterial, antioxidant, and immunomodulatory effects in various mammalian species (Mastinu et al. 2019; Kraljevic Pavelic et al. 2018; Valpotić et al. 2017). However, in the absence of direct measurements of oxidative and cellular damage markers in the present study, these interpretations should be considered hypotheses rather than confirmed mechanisms, consistent with previous studies indicating that zeolite supplementation may improve sperm quality without changes in oxidative biomarkers (Mohammed et al. 2019; Mohammed et al. 2021).

A statistically significant improvement in post‐thaw sperm motility was observed in the 2% and 3% CLP groups compared with the control group. Although the increase from approximately 40% to 45% motility appears numerically modest, even small improvements in post‐thaw sperm quality may contribute to enhanced reproductive outcomes in canine artificial insemination protocols. Previous studies have similarly demonstrated that various extender additives and antioxidants can yield incremental but meaningful improvements in cryopreserved canine semen quality (Monteiro et al. 2009; Ogata et al. 2015). Therefore, the present results indicate that CLP may provide a supportive effect on sperm preservation efficiency comparable to other commonly used supplementation strategies in canine semen cryopreservation.

To our knowledge, this is the first study to evaluate CLP supplementation in canine semen cryopreservation and demonstrates its effects during dilution, equilibration, and post‐thaw stages. The evaluation included comprehensive spermatological parameters (motility, membrane integrity, viability, abnormality, and acrosome damage), together with integrated oxidative stress indices (TAS, TOS, and OSI), allowing a broad assessment of semen quality under cryopreservation conditions. While the results indicate a beneficial role of moderate CLP supplementation, future studies should further investigate these findings by including additional biochemical endpoints such as lipid peroxidation markers and apoptotic indicators to better characterize the underlying biological pathways. In addition, fertility trials following artificial insemination would be valuable to confirm the practical reproductive relevance of CLP supplementation in canine breeding programmes.

## 5 Conclusion

The results of the present study indicate that following cryopreservation and thawing, supplementation with 2% CLP provided the most suitable post‐thaw sperm quality in dogs, whereas the poorest outcomes were observed in the 4% CLP group. The 2% CLP group showed the most balanced oxidative profile, characterized by higher TAS and lower TOS and OSI values, indicating improved overall redox status compared with the other experimental groups. In contrast, both low (1%) and high (4%) concentrations were associated with less favourable oxidative stress parameters, supporting a dose‐dependent effect of CLP supplementation.

In conclusion, 2% CLP appears to be the most effective concentration for improving post‐thaw sperm quality and maintaining oxidative balance in canine semen cryopreservation. Future studies incorporating in vivo artificial insemination trials are warranted to confirm the reproductive outcomes and practical applicability of CLP supplementation in canine breeding programmes.

## Author Contributions

**Nurdan Coşkun**: supervision, writing – original draft preparation, investigations, data curation, conceptualization, visualization, methodology, software. **Fikret Karaca**: investigations, validation, software.

## Funding

This study was supported by the HATAY MUSTAFA KEMAL UNIVERSITY BAP unit with project number 22.GAP.024.

## Ethic Statement

The study was approved by the Scientific Ethics Committee of Hatay Mustafa Kemal University (approval No. 2022/03‐05) and conducted in accordance with relevant guidelines for animal care and use.

## Conflicts of Interest

The authors declare no conflicts of interest.

## Data Availability

Raw data that supports the findings of this study are available from the corresponding author upon reasonable request.
